# Does financial crisis change the relationship between bank development and economic growth? Evidence from US states

**DOI:** 10.1371/journal.pone.0267394

**Published:** 2022-04-21

**Authors:** Fang Li, Sheng Zhang

**Affiliations:** 1 School of Intellectual Property, Nanjing University Science & Technology, Nanjing, Jiangsu, China; 2 School of Public Policy and Administration, Xi’an Jiaotong University, Xi’an, Shannxi, China; Georgia State University, UNITED STATES

## Abstract

This paper investigates the causal relationship between bank development and economic growth in the US before and after the financial crisis in 2008. Based on the quarterly panel data of US states for 2002Q1-2012Q4, this paper uses two-step system GMM method to test the long-term and short-term impact of bank on growth. In addition, panel Granger causality test is used to verify the causality between bank and growth in different periods. The study finds that banks have played a significant role in the long-run economic growth process and there exists a bidirectional causality between bank and growth among US states. In the short term, the financial crisis has corrected the short-run relationship between bank and growth. The relationship between bank and growth has changed from one-way causality before the crisis to two-way causality after the crisis. Before the crisis, economic growth promotes bank credit, but bank development does not promote growth. After the crisis, banks play the role of economic driver through credit, savings and services, and economic growth drives bank savings and scale expansion. The financial intermediary service functions of banks, especially credit to private sector and absorbing savings to transfer loans, determine the role of bank development in economic growth. Policy makers should support bank development in the long run to establish a mature financial intermediary system and promote regional economic development.

## 1. Introduction

A large number of studies have proven that financial intermediary expansion induced by technological progress and deregulation significantly promotes economic growth [1–4]. However, it is unclear whether this relationship can be maintained or not in the event of financial crisis. Existing studies fail to explain the short-term impact and causal relationship between bank and growth during the financial crisis and the impact of the financial crisis on this relationship. This paper attempts to address this gap by studying the impact of the 2008 financial crisis on the relationship between bank development and economic growth in US states.

The past 2008 financial crisis has provided a valuable case for the study of banking and growth. The lower long-term interest rate and the financial innovation of mortgage-backed securitization had exacerbated the deterioration of bank loan standards. The proportion of bank housing mortgage loans and mortgage-backed securities held by banks in bank loans and assets continued to increase. When house prices entered the downward channel in US, the phenomenon of subprime mortgage default continued to increase, and then subprime mortgage crisis occurred in 2007. Speculative investments in the subprime mortgage market linked to mortgage-backed securities worsened the crisis [5, 6]. As a result, the whole economy was badly affected.

The financial crisis led to bank failures and economic recession at the same time. In the fourth quarter of 2008, the US GDP reached 14.5 trillion and exhibited negative growth for three consecutive quarters. After the outbreak of the financial crisis, more than 300 banks closed down. Newly established banks and banks that expanded on a large scale before the crisis were more fragile during the crisis, especially those in areas where the real estate market was in serious distress [7]. The failure of a great quantity of banks led to the withdrawal of banks from the money market loans. Similarly, the total assets of financial intermediaries (total assets of American commercial banks and savings institutions, data from the FDIC) exhibited a downtrend of four quarters, from 13.8 trillion in 2008Q4 to 13.1 trillion in 2009Q4. With the time series data before and after the financial crisis, it is easy to reveal that the variance trends in the GDP and financial development are basically the same.

The existing literature mainly focuses on the impact of the financial crisis on the survival of banks and their financial intermediary service functions (such as savings and investment). Affected by the financial crisis, although the excess reserves of banks were sufficient, more than $2 trillion, bank credit tightened in response to the uncertainty caused by falling house prices and mortgage defaults. During the financial crisis, the loan growth rate of US banks was lower than that before and after the crisis [8]. Banks, especially those whose regulatory ratings were downgraded during this period, saw a sharp decline in loans to small and medium-sized enterprises [9]. With the financial crisis affecting the whole economic development, the American public’s trust in banks had decreased, the function of banks to absorb and mobilize savings had been weakened, and a large amount of savings had been deposited in credit cooperatives [10]. It is generally believed that the banking industry triggered the financial crisis, but there is still a gap in the understanding of the causal relationship between banking and economic growth before and after the crisis.

The objective of this paper is to study the impact of the financial crisis on the relationship between bank development and economic growth. First, verify the significance and causal direction of the relationship between bank development and economic growth, and provide state-level evidence for the literature research on the relationship between them before and after the financial crisis. Second, explore the changes in the relationship between bank development and economic growth in different periods of the financial crisis, reveal the short-term relationship between bank development and economic growth under the influence of the financial crisis, and provide a new explanation for the outbreak of the crisis and its impact. Third, compare the long-term and short-term interaction between bank development and economic growth before and after the financial crisis, clarify the impact mechanism and path, and provide empirical basis for relevant policy-making.

In order to attain the above objectives, this paper uses the quarterly panel data of US States from 2002Q1 to 2012Q4 to test the causal relationship between bank development and economic growth. First, as described by King and Levine [1], this paper uses a two-step system GMM method to examine the effect of bank development on growth. Second, this paper uses the panel Granger causality test method developed by Dumitrescu and Hurlin [11] to test the direction of causality between them. Third, this paper investigates the relationship between bank and growth in a complete economic cycle and will provide regional empirical evidence of long-run relationship on bank and growth. Finally, by dividing this period into three parts, this paper examines the short-term relationship change among sub-periods, which further allows us to compare the difference between long-run and short-run relations and to analyze the influence of the finance crisis.

This paper finds a significant relationship between financial development and growth in a complete economic cycle covering the 2008 financial crisis. Financial intermediary development has promoted local economic growth, and there is bidirectional causality between them throughout the entire period. The results become more complicated in the three sub-periods and show a change in relationship during each period. Economic growth pulled financial intermediary expansion before the crisis. In the post-crisis period, economic recovery drove the development of financial intermediaries, and the financial sector helped restore stable economic growth.

This paper makes three primary contributions to the literature. Firstly, it comprehensively analyzes the short-term and long-term causality between bank development and economic growth in precrisis, postcrisis and the full period, and points out the relationship between bank development and economic growth interacts with the financial crisis. The one-way causal relationship from economic growth to bank development triggered the financial crisis, and the crisis restored the relationship between bank and growth to a two-way causal relationship. This can be seen as the self-healing of an economy with a mature financial system. Secondly, it systematically analyzes the relationship between financial functions of banks, including intermediary services, credit, savings, and economic growth before and after the financial crisis, and finds that different functions play significantly different roles in the relationship between banking and growth. Credit function is an important path to maintain the long-term relationship between bank and growth, and bank intermediary services and savings function are important paths to restore the mutually promoting relationship between bank and growth after the crisis. Thirdly, it demonstrates the interactive relationship between three indicators of bank development and economic growth before and after the financial crisis. These indicators and their relationship with growth can provide reference for the study of state-level economy and bank development.

The remainder of the paper is as follows: Section 2 presents a brief review of the literature and discusses the relationship between financial development and economic growth. Section 3 introduces the econometric methodology and describes the variable definitions and data set used. Section 4 presents the main empirical results, and Section 5 discusses the results obtained. Section 6 concludes the paper.

## 2. Literature review

The issue of the relationship between financial intermediary development and economic growth has been widely debated in the economics and finance literature.

A great many of literatures emphasize the important role of bank development in the process of economic growth theoretically and empirically. Gurley and Shaw [12] note that a mature financial market could accelerate economic growth by mobilizing savings and promoting investment. Patrick [13] summarizes two modes of bank supporting economy: the first are supply-leading patterns wherein financial services run ahead of market demand, especially the demand of entrepreneurs, and promote and motivate entrepreneurial activities; the second are demand-following patterns wherein services of financial intermediaries appear to adapt and support the demand of economic development. Some subsequent studies confirmed that financial development is essential for growth and that government regulations on the financial system, such as interest ceilings and loans to non-production departments, would restrict financial development [14]. Therefore, financial liberalization is conducive to economic growth, especially for underdeveloped countries. In addition, endogenous growth theory considers the financial market as a response to imperfect market mechanisms that will boost growth. Pagano [15] shows an AK growth model and indicates that a steady state of growth depends on the portion of savings turned to investment in the financial sector. Levine [16, 17] presents five services, for example, including producing information and allocating capital, monitoring firms, and exerting corporate governance, risk amelioration, pooling of savings, easing exchange, etc., through which financial intermediary institutions improve information and trade friction, then expedite the long-run economic growth. Regarding the issues of the relationship, early studies have shown that financial development can promote economic growth by examining the significance of the regression coefficient [18, 19]. Levine et al. [3] determine national legality as an effective instrumental variable for making this relationship clear. Beck et al. [20] find that financial development has a positive effect on total factor productivity growth with the GMM method. Zhang et al. [21] study the development experience of 286 cities in China and find a positive role of financial intermediary development on growth. Yeh et al. [22] use the PMG estimate method along with panel data from 40 countries between 1960–2006, and the results show that financial structure is significantly and positively correlated with growth and volatility.

Meanwhile, some studies doubt the vital role of financial development. Existing literature overemphasizes the impact of the financial sector on growth [23–25]. Finance is not an important factor in the process of economic growth; instead, output growth would increase the demand for financial services and have a positive effect on the financial sector. This view that financial development promotes economic growth is inconsistent with the recent development experiences of some countries, and excessively fast deregulation may increase the risk of financial collapse and future economic recession. Rioja and Valev [26] point out that the relationship between financial development and growth may be non-linear. Hasan et al. [27] use provincial-level data in China and find that the regression coefficients are not significant or negative. Wu et al. [28] evaluate the dynamic influence of finance in the economic growth process, and the results show a negative influence on long-run outcome and non-significant influence in the short-term. Rafindadi and Yusof [29] use Kenya’s time series data 1980–2011 to test the impact of financial development on economic growth, and find that financial development will not have an impact on Kenya’s GDP.

It can be seen that the theoretical debate mainly focuses on the economic recession risk brought by the financial crisis and the strength of bank supervision. Due to the differences of measurement methods and research samples, the analysis results of empirical research are different and inconsistent, including positive impact, negative impact, nonlinear impact and no significant impact. It is worth mentioning that most studies basically agree with the importance of how financial development effects economic growth.

With the deepening of theoretical and empirical debate and the continuous development of measurement technology, recent studies try to solve this debate by analyzing the interaction and distinguishing between long-term and short-term effects.

Interactional effect theory underlines the mutually positive influence between bank development and growth [30]. As economic growth increases the demand for financial services, the presence of financial intermediaries could reduce the cost of financing and generate growth. Empirical research mainly uses Granger causality test, VECM model, ARDL and other methods to test the direction and significance of causality and study the interaction between bank development and economic growth. Regarding issues on the direction of causality, some studies show bidirectional causality between finance and growth [31–33]. However, some papers have completely different results. Handa and Khan [34] study a sample of 13 countries at different stages of development and find bidirectional or one-way causality from growth to finance. Hassan et al. [35] examines the short-run relationship in most regions of the world and indicate one-way causation from growth to financial development in Africa and East Asia. Hasan and Barua [36] investigate the long-run relationship in South Asian countries and there exists only a causal relationship between financial development and growth.

In order to further refine the analysis of impact mechanism, recent studies distinguish the long-run and short-run relationships between bank development and growth. Loayza and Ranciere [37] use the ARDL method to estimate the two relations simultaneously and find a long-run positive correlation but a negative correlation in the short run. Bangake and Eggoh [38] estimate these two points using DOLS method and VECM model and determine the bidirectional causality in the long-run. However, in the short run, there exists one-way causation from growth to financial development in high-income countries and no significant causality in low and middle-income countries. Rafindadi and Yusof [39] examines the short-term and long-term impact of financial development on economic growth in Nigeria from 1980 to 2011, and finds that financial development promotes economic growth in the long and short term. Pradhan et al. [40] test the interactions between innovation, financial development, and economic growth in 49 European countries 1961–2014 and find that financial development and innovation are both causal factors of economic growth in the long-run.

According to the research on the impact of the financial crisis, the existing literature believes that the financial crisis will weaken the positive role of bank development on economic growth [37, 41]. Accompanied by the process of financial liberalization, financial depth has a long-term positive effect on economic growth, but it will weaken over time because of the frequent occurrence of bank crises [42]. Finance fluctuations and systemic banking crises would decrease the level of real output growth even under a mature financial system. Rafindadi [43] studies the currency crisis in Nigeria and finds that the currency growth rate and domestic credit growth rate lead to the intensification of the crisis.

To sum up, the existing research has achieved fruitful results in the relationship between bank development and economic growth. There is an interaction between bank development and economic growth. The empirical literature based on different data samples draws different conclusions. When the financial crisis occurs, on the one hand, banks provide financial intermediary services to reduce information and market friction and promote economic growth; On the other hand, the spread of the crisis will lead to economic depression. There is a gap in the existing research on the relationship between bank development and economic growth during the financial crisis. First, the existing research does not analyze the causal relationship between bank development and economic growth before and after the crisis, and does not study the relationship between the change of causal relationship and the financial crisis. Second, the existing research ignores the refinement of the relationship between bank development and economic growth from the perspective of bank financial intermediary service function, and does not study the change of the action path between bank and growth before and after the financial crisis.

## 3. Methodology and data

### 3.1 Econometric methodology

In this section, the dynamic panel sys-GMM method and panel Granger causality test are used to examine the relationship between bank development and economic growth in US states.

Early finance-growth empirical literature estimates augmented growth regression with the OLS method and cross-country data. However, due to sample selection, data frequency, proxy variables and method limitations, the conclusion of cross-section or OLS regression would be unreliable [40, 44]. To eliminate estimation bias, many studies choose to add control variables, use fixed effects regression, introduce instrumental variables or dynamic panel estimates, and so forth [20]. The most effective measure is the dynamic panel GMM method, as it avoids cross-sectional data regression bias, allows bank and growth variables to be obtained using instrumental variables, effectively manages the endogeneity problem of bank development proxy variables, and obtains a more precise estimator.

Therefore, this paper employs the dynamic panel sys-GMM method to examine the relationship between bank development and economic growth in US states. The dynamic panel sys-GMM regression can be expressed as follows:

$$Growth_{i,t}=\alpha +\beta_{1}Growth_{i,t-1}+\beta_{2}{\left[Bank\right]}_{i,t}\;+\beta_{\text{3}}Z_{i,t}+\mu_{i}+\lambda_{t}+\varepsilon_{i,t}$$ (1)

$$Growth_{i,t}=\alpha +\beta_{1}Growth_{i,t-1}+\beta_{2}{\left[Bank\right]}_{i,t}\;+\beta_{\text{3}}{\left[Bank\right]}_{i,t-1}\;+\beta_{\text{4}}Z_{i,t}+\mu_{i}+\lambda_{t}+\varepsilon_{i,t}$$ (2)

where the dependent variable *Growth* is the economic growth rate, *Bank* takes bank indicators, and *Z* takes each of the control sets such as initial income level, unemployment, and inflation. Banks play a significant role in economic growth if coefficient β_2_ is significant and positive. It is useful to bring in a lagged dependent variable as an instrumental variable as this reduces the possibility of estimation bias, and in reality, current economic growth is affected by lagged growth. Eq 2 adds lagged financial indicators to examine whether bank development could predict economic growth: if coefficient β_3_ is significant and positive, bank development will not only follow economic growth but will also promote it.

For the panel Granger causality test, three measures are used. First, a VECM model is built based on instrumental variable estimation [38] but without consideration of sample heterogeneity and cross-sectional dependence. Second, the SUR estimator was used with the Wald test [35]. It solves the cross-sectional dependence problem of sample individuals, but this test must move on to simulation to obtain a critical value. Finally, Dumitrescu and Hurlin [11] develop a new Granger causality test for heterogeneous panel samples that can manage the problem of sample heterogeneity and cross-sectional dependence. Therefore, this paper uses this new Granger causality test to examine the relationship between bank development and economic growth in US states.

### 3.2 Variables

This section defines variables, including dependent variable, independent variables and control variables.

#### 3.2.1 Dependent variable

The most common indicator of economic growth is the GDP growth rate or per capita GDP growth [33, 36]. Furthermore, to capture the channel of the financial intermediary effect on growth, some studies use other indicators, such as total factor productivity, the rate of physical capital accumulation, per capita income growth and the private savings rate [20]. However, the US does not publish quarterly GDP data in state-level economies. Refer to the research of Pan and Wang [45], this paper uses the per capita income growth rate.

#### 3.2.2 Independent variable

Existing papers point out the financial intermediary service functions of banks, but data limitations lead many studies to prefer to measure the scale, productivity, and efficiency of financial intermediaries or markets. King and Levine [1] design indicators measure financial depth, intermediary functions of financial institutions, and asset allocation in the financial system. Their indicators are not suitable for data at the state level. Following the devise thinking of the country level, this paper designs three indicators, to comprehensively measure the development level of banks in US states. These indicators design as follows:

1. The Depth of financial intermediary services is measured by bank deposit liability. Based on the hypothesis that the larger the financial intermediary is, the more financial services they would offer, many studies use M1 or M2 as the financial development indicator [46, 47], measuring the currency degree of an economy. However, undeveloped finance may also result in a high currency degree (M1/GDP), and a low currency degree may be the result of a mature financial system [48]. Gross currency refers to the present service offering ability of the financial sector, rather than the real services offered [49], and the increase in the general currency rate refers to income-related rise in currency rather the bank deposit liability [50]. Therefore, for greater precision, this paper uses the ratio of bank deposit liability to total assets to measure the depth of financial intermediary services, and bank deposit liability = cash+ current deposit.
2. Loans to the private sector measure asset allocation service in the financial system. Compared to extending credit to the public sector, banks loan to firms along with more financial services will lead to a larger outcome increase [1]. Banks will be more rigorous when credited to the private sector, and investment quality improves. Refer to the studies of Odhiambo [46], Cheng and Degryse [51] and Rafindadi and Ozturk [52], this paper uses the ratio of loans to the private sector to total loans to measure asset allocation function. Loans to the private sector = total loans-government and public sector loans- loans between banks.
3. Total deposit measures the function of banks to mobilize and absorb savings. Financial intermediaries can mobilize and pool savings and promote economic growth through capital accumulation and further investment. Deposit converting to investment is the direct channel through which the financial sector affects the economy, and higher deposits could stimulate investment and growth [15]. Refer to the studies of Cheng and Degryse [51] and Ang and McKibbin [53], this paper uses the ratio of total deposits to income to measure savings service function.

#### 3.2.3 Control variable

Most studies tend to add more control variables to increase the precision of regression. This paper collects panel data at the state level, and common variables such as education level, imports and exports, and government expenditures are not applicable. This paper then chooses the following four variables as the control variables:

1. initial income level. Adding the initial income level to the model can control the impact of different initial development levels on economic growth and effectively control growth convergence [51]. This paper use 2001 year-end per capita original income of US states as the initial development level because the sample size is 2002Q1-2012Q4.
2. unemployment. The unemployment rate, as another index of economic development, is closely related to growth and financial development [27, 45]. The quarterly unemployment rate comes from averaging three months of unemployment in each of the states.
3. inflation rate. Inflation rate is also an indicator to measure the macroeconomic situation, which can control the impact of regional macro environment on economic growth [45, 46]. This paper uses the Consumer Price Index (CPI) to control for inflation.
4. national monetization level. Monetization level is an important indicator to measure the financial depth of a country, and also affects the financial service capacity of banks in states [47, 54]. To more precisely estimate the relationship between regional financial indicators and growth, this paper uses the American national monetization level (M2/GDP) to control for national monetary policy and the degree of national financial service capacity.

### 3.3 Data

#### 3.3.1 Data description

This paper collects the panel quarterly data of 51 US states over the period 2002Q1–2012Q4. The data of indicators for the development of financial intermediation are from the Federal Deposit Insurance Corporation (FDIC) bank database at the state level. The data for the variables of growth, initial income level, and US quarterly GDP are from the US Bureau of Economic Analysis. The data for the variables of unemployment and CPI are from the US Department of Labor Bureau of Labor Statistics. Quarterly data for US M2 is from the Federal Reserve. Due to data limitations, we use the regional data as the variable CPI, which means that all the states in the same region have the same value for the variable.

Table 1 presents the descriptive statistics and their correlations for the dependent variable, financial indicators and control variables. There is considerable variation across states.

**Table 1:** Data description statistics and correlations.

Variable		Mean			SD			Min			Max	
Growth		1.075			1.314			-7.325			11.727	
Depth		0.641			0.133			0.075			0.865	
Loans		0.872			0.060			0.597			0.991	
Deposit		2.208			6.667			0.039			92.007	
Initial		10.176			0.135			9.929			10.554	
Unemployment		6.163			2.159			2.3			14.133	
Inflation		3.221			0.277			2.722			3.834	
Monetization		0.542			0.044			0.494			0.634	
	Growth		Depth	Loans		Deposit	Initial		Unemployment	Inflation		Monetization
Growth	1											
Depth	0.029		1									
Loans	0.042**		0.042**	1								
Deposit	0.063***		-0.451***	0.179***		1						
Initial	-0.021		-0.136***	0.119***		-0.073***	1					
Unemployment	-0.162***		0.104***	0.155***		-0.051**	-0.066***		1			
Inflation	-0.065***		0.190***	0.006		-0.162***	0.084***		0.431***	1		
Monetization	-0.155***		0.198***	0.182***		0.083***	0.000		0.653***	0.761***		1

Note: * Indicates that the coefficient is significant at the 10% threshold.

** Indicates that the coefficient is significant at the 5% threshold.

*** Indicates that the coefficient is significant at the 1% threshold.

#### 3.3.2 Data test

In this section, the panel unit root test and panel co-integration test are used to examine the stationarity and cointegration relationship of data.

This paper employs three tests, the Levin-Lin-Chu test (LLC), Im-Pesaran-Shin test (IPS), and Choi test (Fisher typed fuller) to test the stationarity of the data. Section relation of panel data would reduce the effectiveness of tests and thus, each data point will subtract its average cross section. Table 2 shows the results of these tests, with every variable being stationary. The variable CPI misses the IPS test, but it remarkably refuses the null hypothesis that a unit root exists under the LLC test and Choi test, and it is predicated to be stationary. The variable FIN consists only of time series data without cross-sectional characteristics; it rejects the null hypothesis at significance levels of 5% under the LLC test and is thus stationary.

**Table 2:** Results of panel unit root tests.

variable	LLC Test	IPS Test	Choi Test	Stationarity
Growth	-36.1630***	-36.7761***	719.3951***	stationary
Depth	-2.4747***	-1.6835**	303.7052***	stationary
Loans	-1.4941*	-2.8953***	287.5620***	stationary
Deposit	-2.5492***	-3.3263***	130.7674**	stationary
Unemployment	-4.2348***	-2.9431***	271.2926***	stationary
Inflation	-6.2815***	-0.1289	249.2020***	stationary

Note: 1. LLC Test presents adjusted t value, IPS Test presents *W*_*t*-*bar*_ statistics with residual sequence correlation, Choi Test uses inverse chi-square test because of the sample T<N.

2. The LLC test and IPS test were both run under minimizing AIC conditions.

After the panel unit root test, the panel co-integration technique developed by Westerlund et al. [55] is used to test the co-integration relationship between the growth indicator and three financial indicators. The statistics of the between-group and hybrid tests are -6.231 and -42.507, respectively, and both refuse the null hypothesis of no co-integration relationship at the 1% significance level. This indicates that a long-term co-integration relationship exists between the growth indicator and the three financial indicators.

## 4. Empirical results

This section presents the results of sys-GMM regression and panel Granger causality test with the US state data in 2002Q1-2012Q4. Two specification tests are carried out with the system GMM method, the Sargan test and the second-order serial correlation test, to examine the problem of over-identification restrictions and serial correlation of error term.

### 4.1 Results for the full period

In order to test the long-term relationship between bank development and economic growth, the system GMM regression is carried out based on the data of the whole period. The results from full period system GMM estimation are summarized in Tables 3 and 4.

**Table 3:** Full period system GMM estimation (without lagged).

Repressor	1	2	3	4
Growth_t-1_	-0.016	-0.111***	-0.094**	-0.0758***
	(0.044)	(0.038)	(-1.98)	(-8.11)
Depth	5.138***			4.273***
	(0.787)			(4.35)
Loans		20.869***		12.34***
		(3.036)		(7.16)
Deposit			0.005	0.030
			(0.15)	(1.41)
Initial	-0.878	-7.563***	-2.206	-1.033
	(1.284)	(2.010)	(-1.12)	(-1.06)
Unemployment	-0.222***	-0.134***	-0.270***	-0.171***
	(0.035)	(0.037)	(-3.88)	(-7.89)
Inflation	0.331	2.980***	1.653***	1.464***
	(0.388)	(0.491)	(2.89)	(4.17)
Monetization	-0.411	-19.779***	-2.768	-11.55***
	(2.418)	(3.429)	(-0.66)	(-4.88)
cons	7.258	61.888***	21.45	0.716
	(13.130)	(19.934)	(1.07)	(0.08)
*N*	2193	2193	2193	2193
*IV* number	172	172	172	340
AR(2)	0.0133	0.1323	0.1171	0.0535
Sargan Test	1.0000	1.0000	1.0000	1.0000

Note: AR (2), the second-order serial correlation test, and the Sargan test show the p value.

**Table 4:** Full period system GMM estimation (with lagged).

Repressor	1	2	3	4	5
Growth_t-1_	-0.018	-0.115**	-0.094**	-0.098**	-0.099**
	(0.043)	(0.046)	(0.045)	(0.041)	(0.043)
Depth					5.081**
					(2.147)
Depth _t-1_	2.792***			2.593***	-1.749
	(0.882)			(1.116)	(1.479)
Loans					2.517
					(6.377)
Loans _t-1_		19.775***		13.900***	11.931***
		(2.802)		(2.138)	(3.609)
Deposit					-0.045
					(0.028)
Deposit _t-1_			0.023*	0.032***	0.080***
			(0.013)	(0.012)	(0.016)
Initial	-1.559	-6.184***	-2.426	-2.624**	-2.512
	(1.505)	(1.441)	(2.323)	(1.299)	(2.423)
Unemployment	-0.220***	-0.141***	-0.273***	-0.159***	-0.165***
	(0.036)	(0.042)	(0.060)	(0.033)	(0.043)
Inflation	0.333	2.754***	1.639***	1.779***	1.928**
	(0.315)	(0.375)	(0.583)	(0.444)	(0.768)
Monetization	0.147	-17.005***	-3.545	-13.485***	-14.247***
	(3.164)	(3.262)	(3.193)	(2.957)	(4.847)
cons	15.387	48.088***	24.133	15.946	14.456
	(15.382)	(14.970)	(23.502)	(12.678)	(23.840)
*N*	2193	2193	2193	2193	2193
*IV* number	170	170	170	334	334
AR(2)	0.0218	0.2749	0.1237	0.1706	0.0814
Sargan Test	1.0000	1.0000	1.0000	1.0000	1.0000

Table 3 shows the results of full period system GMM estimation without lagged independent variables. Model 1–3 display the results including three bank development indicators in the regression. Model 4 confirms the previous results for bank development indicators. Model 1–2 show that two bank development indicators, Depth and Loans, are significantly positive at the 1% level of significance, and the influence coefficients are 5.138 and 20.869, respectively. Model 3 shows that the coefficient of Deposit is positive but insignificant. The regression results of model 4 are consistent with those of model 1–3. This shows that bank development promotes economic growth through credit and the provision of intermediary services. Most regressions pass the Sargan test and second-order serial correlation test at the usually acceptable level of significance, indicating that the regression results are credible. In Model 1, the coefficient of Depth does not pass the serial correlation test.

Table 4 shows the results of full period system GMM estimation with lagged independent variables. Model 1–3 display the results including lag value of three bank development indicators in the regression. Model 4 confirms the previous results for lagged bank development indicators. Model 5 adds the current value of bank indicators on the basis of model 4. According to model 1–3, all three bank development indicators, Depth_t-1_, Loans_t-1_, and Deposit_t-1_, are significantly positive, and the influence coefficients are 2.792, 19.775 and 0.023, respectively. The results of model 4 are consistent with those of model 1–3. This means that in the long run, bank development could predict economic growth.

Model 5 in Table 4 shows the results of Eq 2 in Section 3.1. The coefficient of Depth is significantly positive, which is 5.081, while the coefficient of its lagged variable, Depth_t-1_, is negative and insignificant. This means that the depth of financial intermediary services has a positive role in promoting growth but cannot be regarded as a predictive indicator for economic growth. The coefficient of Loans is positive but insignificant, while the coefficient of the lagged variable Loans_t-1_ is significantly positive, which is 11.931. This indicates that the lagged degree of credit to the private sector seems predictable for growth. The larger the scale of credit to the private sector, the faster the economic growth in the next year. The coefficient of Deposit is insignificant, while the coefficient of Deposit_t-1_ is significantly positive, which is 0.080. The absorption of savings by banks will not directly promote economic growth. Savings need to be transformed into investment to drive economic development. Therefore, the positive effect of bank savings on economic growth has a certain lag. Higher deposit levels will lead to faster economic growth in the next year.

The results presented in Tables 3 and 4 show that bank development exerts a long-run positive impact on economic growth. Furthermore, all three financial service functions of banks have played a significant role in promoting economic growth. It means that the development of banks not only accompanies economic growth, but also promotes economic growth. The coefficient of lagged growth Growth_t-1_ is significantly negative, which means that growth is conditionally convergent in US states, which is consistent with the endogenous growth literature [56]. That is, a low initial level will have a high rate of growth, and the steady state of growth is related to financial development. Among the control variables, Monetization is significantly and negatively correlated with economic growth. Excessive expansionary monetary policy decreases interest rates and encourages investors to enter the real estate market, especially the subprime mortgage market. Banks transfer the risk of default to securities buyers through the capital market, which exacerbates the problem of adverse selection and moral hazard incentives in the loan market and accelerates crisis outbreaks and diffusion. This shows that the quantitative easing policy is not as good as desired, and it brings negative effects in this crisis. Unemployment has a significant negative impact on economic growth. There is a significant positive correlation between Inflation and economic growth.

### 4.2 Results for the sub-periods

To capture the short-run relationship between financial development and growth at different stages of the economic cycle, especially the effect of the financial crisis on the relationship, this paper divides the whole period into three parts: term before the crisis (2002Q1-2007Q2), term during the crisis (2007Q2-2009Q4), and term after the crisis (2009Q4-2012Q4).

The results of sub-period system GMM estimations are summarized in Tables 5 and 6, and most regressions, excepting model 6, pass the Sargan test and second-order serial correlation test. It shows that these regression models do not have the problems of over identification and residual autocorrelation.

**Table 5:** Sub-period system GMM estimation (without lagged).

Sub-period	2002Q1-2007Q2			2007Q2-2009Q4			2009Q4-2012Q4		
Repressor	1	2	3	4	5	6	7	8	9
Growth_t-1_	-0.219***	-0.213***	-0.220***	-0.081	-0.096	-0.0620	-0.273***	-0.31***	-0.235***
	(0.072)	(0.075)	(-3.17)	(0.082)	(0.139)	(-1.04)	(0.071)	(0.082)	(-2.86)
Depth	3.02**			4.001**			1.342		
	(1.228)			(1.912)			(4.049)		
Loans		3.201			7.006			7.848	
		(3.308)			(32.367)			(9.898)	
Deposit			-0.044*			0.126*			-0.071***
			(-1.76)			(1.94)			(-2.95)
Initial	1.499	-2.609*	-1.195	-0.761	-6.250	-4.295	-5.537*	-0.248	9.622
	(1.413)	(1.461)	(-0.83)	(2.044)	(3.969)	(-1.29)	(3.006)	(2.939)	(1.39)
Unemployment	-0.019	0.069	-0.131	0.528***	0.569***	0.312***	-0,641***	-0.861***	-0.874***
	(0.136)	(0.102)	(-1.24)	(0.082)	(0.150)	(2.69)	(0.192)	(0.190)	(-5.18)
Inflation	1.831*	2.574**	1.363***	2.205***	2.237**	1.889*	-1.035	-0.846	-7.387**
	(0.984)	(1.113)	(3.00)	(0.622)	(1.029)	(1.83)	(1.996)	(2.343)	(-2.49)
Monetization	6.956	-3.291	7.097	-55.72***	-59.85***	-40.42***	-13.25	-23.96*	16.60
	(5.019)	(6.588)	(1.08)	(4.893)	(12.025)	(-5.33)	(12.967)	(14.117)	(0.95)
cons	-24.402	19.000	6.804	25.485	81.079	58.65	73.763	21.401	-73.13
	(12.870)	(12.941)	(0.45)	(22.390)	(31.099)	(1.55)	(30.711)	(28.614)	(-1.05)
*N*	1071	1071	1071	510	510	510	612	612	612
*IV* number	84	84	84	76	76	76	48	48	48
AR(2)	0.9114	0.8427	0.8077	0.0538	0.1366	0.0138	0.4115	0.5016	0.4362
Sargan	0.9915	0.9911	0.9930	0.9621	0.9505	0.0584	0.1493	0.1491	0.1672

**Table 6:** Sub-period system GMM estimation (with lagged).

Sub-period	2002Q1-2007Q2			2007Q2-2009Q4			2009Q4-2012Q4		
Repressor	1	2	3	4	5	6	7	8	9
Growth_t-1_	-0.225***	-0.204***	-0.229***	-0.091	-0.141	-0.0676	-0.259***	-0.282***	-0.267***
	(0.068)	(0.065)	(-3.37)	(0.083)	(0.090)	(-1.08)	(0.074)	(0.075)	(-2.85)
Depth	7.455***			4.389			27.592*		
	(1.738)			(6.736)			(14.548)		
Depth _t-1_	-7.362***			-4.114			-26.825*		
	(2.557)			(6.084)			(14.265)		
Loans		19.551**			-2.881			3.714	
		(8.276)			(21.25)			(11.740)	
Loans _t-1_		-16.60**			11.888			3.910	
		(7.070)			(19.828)			(14.016)	
Deposit			-0.340			-0.0364			-0.162***
			(-1.15)			(-0.13)			(-6.16)
Deposit _t-1_			0.345			0.171			0.079**
			(1.15)			(0.56)			(2.34)
Initial	-0.574	-2.062	-0.693	-2.532	-1.889	-3.402	-3.510	-1.391	0.784
	(1.075)	(1.421)	(-0.54)	(3.143)	(3.207)	(-1.17)	(2.209)	(3.131)	(0.13)
Unemployment	0.064	-0.175	-0.123	0.582***	0.532***	0.318***	-0.579***	-0.628***	-1.062***
	(0.112)	(0.129)	(-1.02)	(0.105)	(0.108)	(2.85)	(0.173)	(0.172)	(-4.41)
Inflation	2.458***	1.898	1.467**	1.480	2.288**	1.892*	0.185	-2.641	-4.749
	(0.921)	(1.180)	(2.33)	(0.923)	(1.141)	(1.75)	(1.976)	(2.634)	(-1.60)
Monetization	2.350	1.361	4.171	-58.03***	-59.76***	-41.43***	-17.445	-9.982	0.692
	(7.815)	(8.260)	(0.63)	(5.626)	(6.850)	(-5.54)	(12.763)	(12.297)	(0.04)
cons	-1.469	14.575	2.820	49.621	34.138	49.87	51.153	29.276	18.63
	(9.757)	(12.902)	(0.22)	(34.115)	(31.748)	(1.49)	(23.898)	(31.247)	(0.29)
*N*	1071	1071	1071	510	510	510	612	612	612
*IV* number	82	82	82	46	46	46	46	46	46
AR(2)	0.7779	0.8067	0.5982	0.0502	0.1183	0.0154	0.7028	0.2853	0.8684
Sargan	0.9845	0.9850	0.9856	0.0988	0.0945	0.0271	0.1102	0.0869	0.0914

Table 5 shows the results from system GMM estimation without lagged independent variables. Model 1–3 show the regression results of the period before the financial crisis. Model 4–6 show the regression results during the financial crisis. Model 7–9 show the regression results in the period after the financial crisis. In the two periods before and during the financial crisis, only Depth has a significant positive effect on economic growth. The impacts are also economically significant, with impact coefficients of 3.02 and 4.001 respectively. It indicates that even during the financial crisis, banks still maintain a positive contribution to economic growth. After the financial crisis, only Deposit has a significant negative impact on economic growth, with an impact coefficient of -0.071. It shows that in the post crisis economic recovery stage, the mobilization of savings by banks is not conducive to economic growth, which contrasts with the view that a well-developed financial sector would generate an increase in the deposit rate and investment and then promote growth. The impact of Loans on economic growth is not significant in all three sub periods. It shows that, unlike the long-term influence, bank credit to the private sector does not promote economic growth in the short term.

Table 6 shows the results of sub-period system GMM estimation with lagged independent variables. Model 1–3 show the regression results of the period before the financial crisis. Before the crisis, the coefficients of Depth and Loans are significantly positive, which are 7.455 and 19.551, respectively. While their lagged variables, Depth_t-1_ and Loans_t-1_, are significantly negative, and the influence coefficients are -7.362 and -16.60, respectively. It means bank development has promoted economic growth during this period. Model 4–6 show the regression results during the financial crisis. During the crisis, each of the three indicators is insignificant, which implies that the financial crisis neutralizes the positive effect of bank development on growth. Model 7–9 show the regression results in the period after the financial crisis. After the financial crisis, the influence coefficient of Deposit remains significantly negative. The influence coefficient of Deposit_t-1_ is significantly positive, which is 0.079. This is consistent with the long-term influence, which means that banks mobilize savings would promote future economic growth.

The results presented in Tables 5 and 6 show that although the short-term relationship between bank development and economic growth before and after the financial crisis is obviously different from the long-term relationship, there is a positive correlation between bank development and economic growth in each period.

### 4.3 Causality analysis

This section uses the panel Granger causality test developed by Dumitrescu and Hurlin [11] to examine the direction of the causal relationship between bank development and growth. Because this test requires a sufficient length of time series, the sample can only be divided into two periods: before the crisis (2002Q1-2007Q2) and after the crisis (2007Q2-2012Q4).

Table 7 presents the results of causality test, which indicate different causality relationships at different periods. In the long-run (2002Q1-2012Q4), there is a significant positive two-way causal relationship between Loans and Growth, with impact coefficients of 3.319 and 9.725 respectively. It shows that there is bidirectional causality between bank and growth, and banks significantly promote growth through credit to private firms. This is consistent with the conclusion of Rafindadi and Yusof [57]. There is a significant positive one-way causality from Growth to Deposit, while a significant negative one-way causality from Growth to Depth. It means economic growth brings deposit increase, but does not bring more supply for financial services of banks.

**Table 7:** Panel Granger causality test.

The direction of causality		2002Q1-2012Q4	2002Q1-2007Q2	2007Q2-2012Q4
Bank→Growth	Depth→Growth	0.766	1.528	15.544***
	Loans→Growth	3.319***	1.003	10.059***
	Deposit→Growth	0.341	0.974	8.051***
Growth→Bank	Growth→Depth	-2.056**	-2.243**	2.056**
	Growth→Loans	9.725***	3.195***	0.725
	Growth→Deposit	3.894***	0.902	2.148**

In the short-run, the causality before and after the crisis occurs exhibits obvious differences. Before the crisis (2002Q1-2007Q2), There is a significant positive one-way causality from Growth to Loans, while a significant negative one-way causality from Growth to Depth. It means economic growth brings more demand for credit, but not more supply for financial services of banks. Therefore, there exists only one-way causality from growth to bank development, which is consistent with Bangake and Eggoh [38]. The positive correlation of bank and growth due to economic growth yields and drives financial intermediary expansion.

After the crisis (2007Q2-2012Q4), there exists significant positive bidirectional causality between Depth, Deposit and Growth, and a significant positive one-way causality from Loans to Growth. After the crisis, the bank’s credit, savings and service depth have promoted economic growth, and economic growth has driven the expansion of bank service depth and savings, that is, there is mutual promotion between banks and growth. That is, after the financial crisis in the US, economic growth drives banks to recover development, while banks make up for the financial crisis impact of the economy and help the economy steadily grow.

## 5. Discussion

Comparing the empirical analysis results for periods of the cycle, this paper finds the mechanism and development logic behind the change of long-term and short-term relationship between bank development and economic growth.

In the long term, bank development can obviously promote economic growth and predict economic growth. This result is consistent with most research conclusions [3, 22, 58]. Furthermore, the result that there exists positive bidirectional causality between bank and growth, which is consistent with the conclusion of Calderón and Liu [32] and Apergis et al. [33]. That is, in the relationship between bank development and economic growth, service driven mode and demand driven mode coexist. Specifically, banks mainly promote economic growth by providing credit services, and economic growth increases credit demand and deposits, and then drives the development of banks.

In the short term, before the crisis, the influence of bank on growth is rather changeable, and even some index effects are negative, which is consistent with Loayza and Ranciere [37]. Causality analysis shows that at this stage there was only positive unidirectional causality from growth to bank credit, and financial innovation resulted in the decrease of lending standards in the US, causing an unprecedented credit expansion. The rapid expansion of the financial intermediary market brought many benefits for investors but also formed a new systemic risk. Causality analysis also shows that bank development has no contribution to economic growth, and economic growth has restrained the expansion of bank intermediary services. This means that there is a mismatch between the demand for financial services of economic growth and the supply of intermediary services of banks, and banks have not provided corresponding services to meet the demand for financial services brought by economic growth. Some financial services of banks seem to be decoupled from economic growth, which brings hidden dangers to the occurrence of the financial crisis. Due to a lack of market regulation measures, the financial crisis eventually occurred. It also provides empirical support for Dell’Ariccia and Marquez’s [59, 60] theory analysis.

After the crisis occurred, compared with other periods, the role of bank development in promoting economic growth has become less obvious. It may be intelligible, on the one hand, that financial crisis leads to banks facing survival crisis and risk loss, and bank intermediary function services are seriously restricted. It is hardly promoting economic growth through capital accumulation and innovation encouragement. On the other hand, the US government started to reform financial regulations after the crisis and put out tougher capital and liquidity restrictions on bank activities. However, the causality test indicates the bidirectional causality between finance and growth, which is different from Rousseau and Wachtel [42]. Although the financial crisis hurts the economy in the short term and weakens the promoting function of bank on growth, once the crisis is under control, the well-developed and mature financial intermediation system will resume development together with the economy.

Comparing the results before and after the crisis, the financial crisis seems to have changed the relationship between bank and growth, from unidirectional causality to bidirectional causality.

But in the long run, the financial crisis has corrected, more accurately, the relationship between banks and economic growth, and restarted the benign feedback mechanism between bank and growth. The decisive factor behind the relationship between bank development and economic growth lies in whether the financial intermediary services of banks meet the financial needs of the development of the real economy or not. After the crisis, financial supervision and securities market risk make banks pay careful attention to the credit demand of production sector and provide financial services to meet the needs of real economy.

## 6. Conclusion

This paper uses the panel quarterly data of 51 US states 2002Q1-2012Q4 and examines the relationship between bank development and economic growth for a complete economic cycle that covers the 2008 financial crisis. Employing the system GMM estimate and panel Granger causality test, the following conclusions are found in this paper.

First, banks have played a significant role in the long-run economic growth process and there exists a bidirectional causality between bank and growth among US states. Therefore, bank development has a long-term positive effect on economic growth, and the damage of the financial crisis to the economy has not affected this relationship. Even during the financial crisis, we should face up to the positive role of banks in economic development.

Second, the financial crisis has corrected the short-run relationship between bank and growth. In the short term, the relationship between bank and growth has changed from one-way causality before the crisis to two-way causality after the crisis. Economic growth always promotes the development of banks, but vice versa. Before the crisis, banks blindly expanded by relying on the so-called innovation of financial products and lost their support for the development of the real economy, leading to the final outbreak of the crisis. After the crisis, banks returned to traditional financial intermediary services under financial supervision and internal risk control, and the relationship between bank and growth resumed mutual promotion.

Third, the financial intermediary service functions of banks, especially private sector credit and absorbing savings to transfer loans, determine the role of bank development in economic growth, and are also important factors affecting the relationship between bank and growth. Before the crisis broke out, the hidden danger was already hidden in the relationship between banks and growth. The financial intermediary services of banks can provide corresponding detection indicators to monitor whether the relationship between banks and growth remains stable or not.

The above conclusions also have important policy and countermeasures significance for policy makers. First, bank development has a long-term role in promoting economic growth. The government should support the development of banks for a long time and guide banks to optimize financial intermediary services to serve the development of the real economy. Second, the government should strengthen the reform of the financial sector and financial regulatory policies, establish a mature financial intermediary system, and improve the ability of banks to control risks and provide financial services. Third, strengthen the dynamic monitoring of the relationship between bank development and economic growth. Design monitoring indicators according to the bank’s financial intermediary service function to improve the bank’s risk prevention and early warning level.

By studying the relationship between bank development and economic growth during the financial crisis, this paper finds the impact of the financial crisis on the relationship between bank development and economic growth, which fills the gap in the existing research on the short-term relationship between bank and growth during the crisis. However, this study also has some limitations. First, the research data of this paper comes from US. The United States has established a mature financial intermediary system with strong anti-risk ability and high level of financial supervision. After the crisis, banks quickly resumed their active role in economic growth. However, whether this conclusion is applicable to other economies, especially developing countries, remains to be further verified. Second, the data studied in this paper is at the state level. Due to the limitation of data availability, the role of banks in promoting economic growth by implementing corporate governance, improving risk and facilitating transactions has not been tested. It needs to be further verified based on more micro or macro data.
